# Aberrant mediastinal mediobasal segmental pulmonary artery in a patient with lung cancer undergoing right lower lobectomy: a case report

**DOI:** 10.1186/s13019-022-01837-3

**Published:** 2022-04-27

**Authors:** Kazuyuki Komori, Hiroshi Hashimoto, Kotaro Yoshikawa, Koji Kameda, Shinichi Taguchi, Yuichi Ozeki

**Affiliations:** 1 Departmant of Thoracic Surgery, National Defense Medical College, 3-2, Namiki, Tokorozawa, Saitama 359-8513 Japan; 2 Department of Thoracic Surgery, Tokorozawa-Meisei Hospital, 5095, Yamaguchi, Tokorozawa, Saitama 359-1145 Japan

**Keywords:** Right aberrant mediastinal pulmonary artery, Lung cancer, Right lower lobectomy

## Abstract

**Background:**

A mediastinal mediobasal segmental pulmonary artery (A7) from the right main pulmonary artery is extremely rare.

**Case presentation:**

We report the case of a 71-year-old woman with an aberrant mediastinal A7 who underwent right lower lobectomy for lung cancer (cT1bN0M0, stage IA2). Preoperative three-dimensional computed tomography (CT) angiography revealed an aberrant mediastinal A7 in the right main pulmonary artery. Right lower lobectomy and mediastinal lymph node dissection were performed. Intraoperatively, A7 was observed between the superior and inferior pulmonary veins and in the front of the lower bronchus near the anterior hilum. The artery was carefully dissected from the caudal side after inferior pulmonary vein dissection. The lung parenchyma, which was within the fissure due to poor lobulation between the middle and lower lobes, was safely divided.

**Conclusions:**

Thoracic surgeons need to carefully evaluate CT angiography or enhanced multidetector CT findings at preoperative conferences and always keep this anomaly in mind.

## Background

The branching of the pulmonary artery, vein, and bronchus varies among individuals [1]. For thoracic surgeons, it is very important to preoperatively analyze the anatomic variations to perform safe lung resection. Here, we report an extremely rare case of a patient with lung cancer with a right mediastinal mediobasal pulmonary artery (A7) who underwent right lower lobectomy.

## Case presentation

**Fig. 1:**
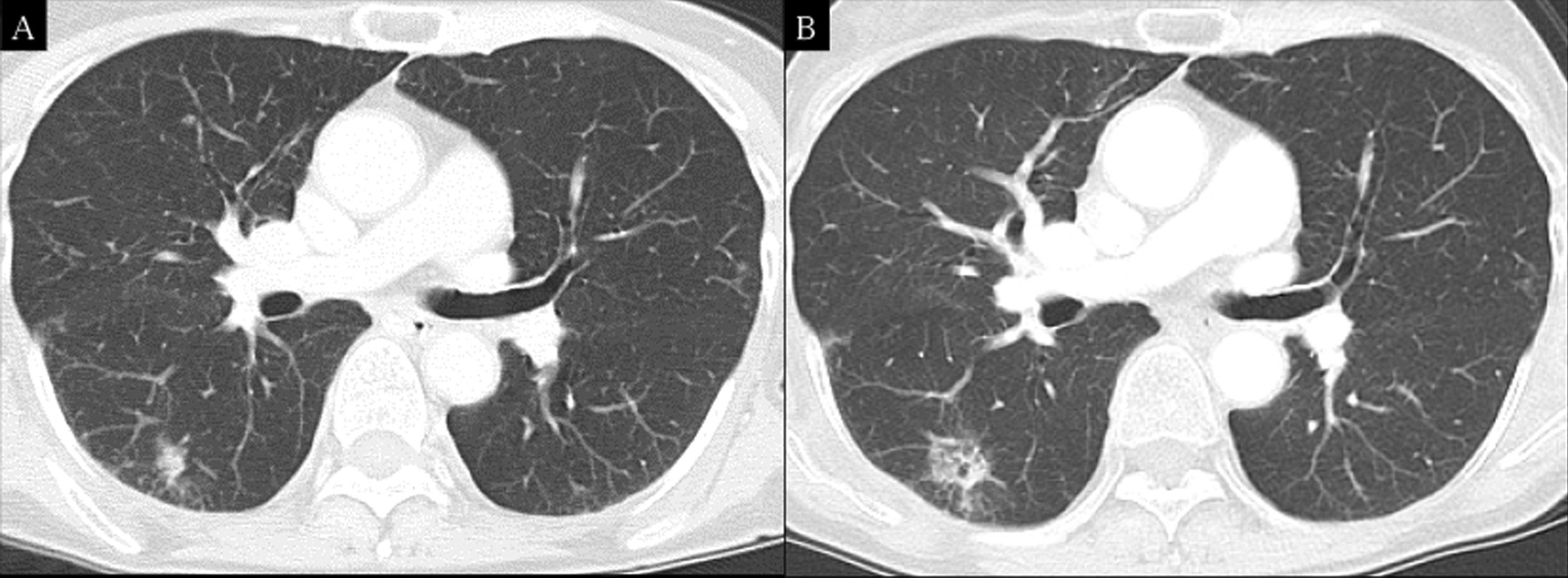
Chest computed tomography. **A** Semi-solid ground-glass attenuation (GGA) in the right lower lobe, **B** a slow-growing GGA in 1.5 years

**Fig. 2:**
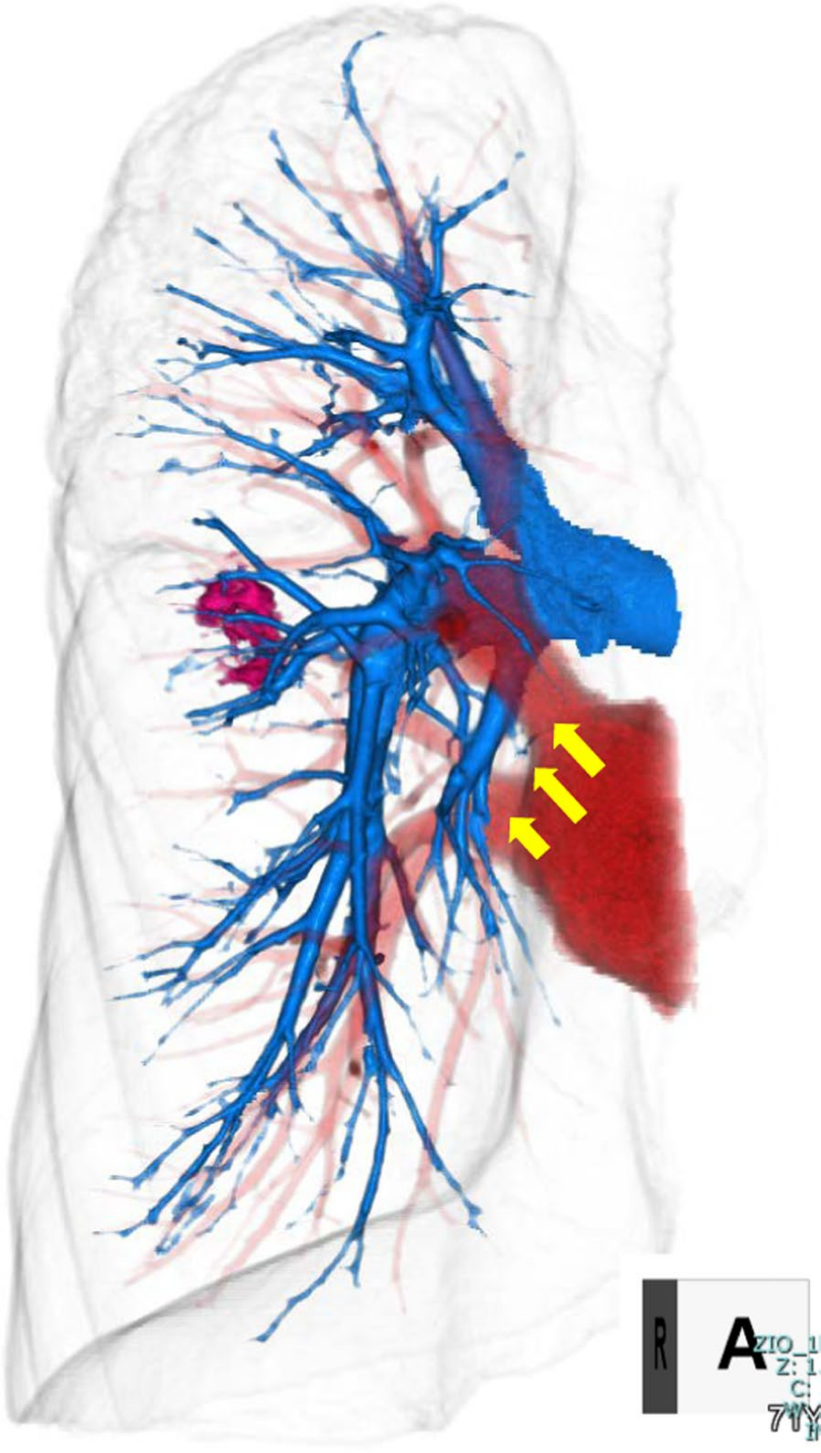
Preoperative three-dimensional computed tomography angiography of the right lung. An aberrant mediastinal A7 (yellow arrow) is seen arising directly from the right main pulmonary artery (blue vessel) on the opposite side of A1 + 2a + 3, the first branch of the pulmonary artery, running between the superior and inferior pulmonary veins (red vessel) and progressing into the medial portion of the right lower lobe

**Fig. 3:**
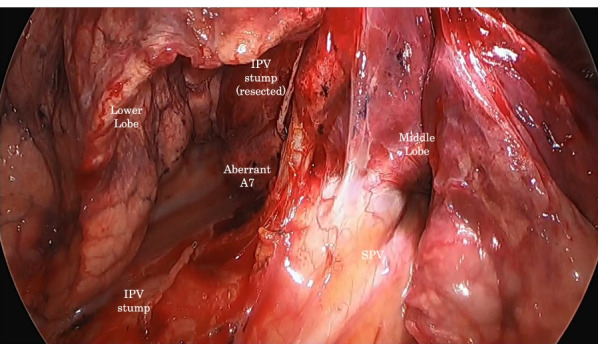
Intraoperative finding from the caudal side after resection of inferior pulmonary vein (IPV). An aberrant A7 is seen running from the hilum between IPV stump and superior pulmonary vein (SPV)

A 71-year-old woman presented to our department with a slow-growing semi-solid ground-glass nodule in the right lower lobe (S6) (Fig. 1) identified on follow-up computed tomography (CT) for oropharyngeal cancer after chemoradiotherapy. No lymph nodes or distant metastases were detected on positron emission tomography/CT. Thus, she was suspected to have primary lung cancer (cT1bN0M0, stage IA2). Preoperative three-dimensional (3D) CT angiography revealed an aberrant mediastinal A7 arising directly from the right main pulmonary artery, running between the superior and inferior pulmonary veins and entering the right lower lobe (Fig. 2). Lung function tests revealed a vital capacity of 2.80 L (115.2% predicted), a forced expiratory volume in 1 s (FEV_1_) of 2.25 L (113.6% predicted FEV_1_), and a diffusing capacity for carbon monoxide of 13.36 ml/min/mmHg (87.3% predicted). Video-assisted posterolateral fifth intercostal thoracotomy was performed with a small 8-cm incision. During surgery, the interlobular fissure between the middle and lower lobes was found to be incomplete, without effusion or adhesion. A hard, elastic tumor measuring 2 cm with slight pleural change was found on the interlobar side in S6a. Aspiration cytology of the tumor revealed a class V adenocarcinoma. As A7 was located at the anterior hilum on preoperative CT, the operative procedure was performed on the dorsal and caudal sides. First, the interlobular main pulmonary artery was exfoliated. Then, A6 and A8–10 were dissected with ligation and autosuture, respectively, after interlobular incision between the upper and lower lobes. Subsequently, the inferior pulmonary vein was dissected after the division of the pulmonary ligament. A7 was identified at the anterior hilum, running between the middle lobe pulmonary vein and the inferior bronchus (Fig. 3). Finally, an interlobar incision between the middle and lower lobes was safely made after A7 dissection with autosuture. The operative time was 153 min, and the total amount of blood loss was 25 g.

The patient received pleurodesis therapy with OK-432 for prolonged air leakage on postoperative day 8. The chest drain was removed on postoperative day 10, and the patient was discharged on postoperative day 13. The final pathological diagnosis was lung papillary adenocarcinoma, pT1b (invasive diameter, 12 mm) N0M0 pStage IA2. There was no evidence of recurrence 6 months postoperatively.

## Discussion and conclusions

Thoracic surgeons often encounter anomalies of the pulmonary arteries, veins, and bronchi. It is highly possible to experience unexpected bleeding from the abnormal pulmonary arteries. This commonly results from the left mediastinal lingular artery, which is the first branch of the left main pulmonary artery. Mediastinal lingular arteries, including the A4+5 type and A4 or A5 type, have been reported in 27.3% of cases [2]. In contrast, Hong et al. reported that the incidence of a mediastinal basal pulmonary artery was 0.05% on both sides [3]. Moreover, in other anatomical pulmonary artery studies, the A7a type (which branches off in front of the inferior pulmonary vein) was observed in 74.8% of cases, A7ab (which branches off on both sides) was in 14.8%, and A7b (which was behind it) was in 4.8% [2, 4]. However, the right mediastinal basal artery has not been reported.

To date, five cases of right mediastinal basal pulmonary artery (Table 1) have been reported in the English literature, all of which were reported in Japan due to the prominence and availability of high-resolution CT. There were two cases of aberrant A7 and one case each of A7a, A6–10, and A7–10. To the best of our knowledge, this is the first reported case of aberrant A7 that independently branched off during right lower lobectomy. Aberrant vessels were identified in all six cases, including our case, in which preoperative multidetector CT and 3D-CT angiography were performed. Intraoperative findings identified poor lobulation in all cases. Operative procedures can be classified depending on whether the interlobular incision between the middle and lower lobes or resection of an aberrant artery was first performed. In three of four cases in which the pulmonary artery in the lower lobe was resected, an interlobular incision was first performed. In our case, we first transected the inferior pulmonary vein with autosuture from the dorsal side for better visualization of the hilum. Then, we exfoliated the aberrant A7 and transected it with autosuture. An interlobular incision was made safely, and the lower bronchus was transected. We encountered no complications during lymph node 7 dissection. After surgery, pleurodesis was required for prolonged air leakage (same as in case 2), but the postoperative course was otherwise uneventful. At present, the formation of a thrombus in the peripheral stump of the pulmonary artery has not been detected, as in other patients.

**Table 1:** Reports of right mediastinal basal pulmonary artery: A review of literature

Case	Journal	Age	Sex	Diagnosis	Location	Size(mm)
1	GTCS/2011 [5]	72	M	Metastasis from rectal cancer	Rt. S7	7
2	Surg Case Rep/2016 [6]	67	M	Lung cancer	NR	NR
3	GTCS/2017 [7]	76	M	Squamous cell carcinoma	NR	65
4	ATS/2020 [8]	74	F	Invasive mucinous adenocarcinoma	Rt.S6-9	73
5	Surg Case Rep/2021 [9]	73	M	Combined small cell carcinoma	Rt. S9	18
6	Our case	71	F	Papillary adenocarcinoma	Rt. S6	25

Case	Preoperative exam	Aberrant	Operative procedure	Operation	Interlobular fissure
1	MDCT angiography	A7	Right basal segmentectomy	VATS	Incomplete
2	3D angiography	A7	Right middle lobectomy	VATS	Incomplete
3	MDCT	A7-10	Right lower lobectomy	VATS	Incomplete
4	Enhanced TSCT	A6-10	Right lower lobectomy	Small thoracotomy	Incomplete
5	CT angiography	A7a	Right basal segmentectomy	VATS	Incomplete
6	3D angiography	A7	Right lower lobectomy	Small thoracotomy	Incomplete

Case	Operative step ①	②	③
1	Interlobular incision	Aberrant A7	
2	Interlobular incision	Not resected aberrant	
3	Interlobular incision	Aberrant A7-10	
4	Interlobular incision	Aberrant A6-10	
5	Abasal + aberrant A7a	Bbasal + V7-10	Intersegmental incision
6	A6 + Abasal	IPV + aberrant A7	Interlobular incision

Case	Operation time (min)	Blood loss (ml)	Complication	Postoperative course
1	NR	NR	None	No rec. at 8POM
2	NR	NR	Air leak	No rec. at 7POM
3	NR	NR	None	NR
4	80	10	None	Adjuvant chemotherapy and no rec
5	144	30	None	No rec. at 1POY
6	153	25	Air leak	No rec. at 6POM

NR: not referred; MDCT: multi-dimension computed tomography; TSCT: thin-slice CT; VATS: video assisted thoracic surgery; IPV: inferior pulmonary vein; POM: postoperative month

It is important to check vessel variation using 3D-CT angiography and discuss the safe operative procedure at the preoperative conference.

In summary, we reported a case of a patient with lung cancer and a mediobasal mediastinal pulmonary artery arising from the main pulmonary artery who underwent right lower lobectomy. Our report suggests that attention should be paid to these anomalies.

## Data Availability

The datasets used and/or analyzed during the current study are available from the corresponding author on reasonable request.

## Abbreviations

3D: Three-dimensional
A7: Mediobasal pulmonary artery
CT: Computed tomography
FEV_1_: Forced expiratory volume in 1 s
